# Longitudinal optical imaging technique to visualize progressive axonal damage after brain injury in mice reveals responses to different minocycline treatments

**DOI:** 10.1038/s41598-020-64783-x

**Published:** 2020-05-08

**Authors:** Chelsea D. Pernici, Rachel K. Rowe, P. Timothy Doughty, Mahboubeh Madadi, Jonathan Lifshitz, Teresa A. Murray

**Affiliations:** 1 Center for Biomedical Engineering and Rehabilitation Sciences, Louisiana Tech University, Ruston, LA USA; 2 Barrow Neurological Institute at Phoenix Children’s Hospital, Phoenix, AZ USA; 3 Department of Child Health, University of Arizona College of Medicine – Phoenix, Phoenix, AZ USA; 4 Phoenix Veterans Affairs Health Care System, Phoenix, AZ USA; 5 Department of Marketing and Business Analytics, Lucas College of Business, San Jose State University, San Jose, CA USA

**Keywords:** Brain injuries, Neurodegeneration, White matter disease, Optical imaging

## Abstract

A high-resolution, three-dimensional, optical imaging technique for the murine brain was developed to identify the effects of different therapeutic windows for preclinical brain research. This technique tracks the same cells over several weeks. We conducted a pilot study of a promising drug to treat diffuse axonal injury (DAI) caused by traumatic brain injury, using two different therapeutic windows, as a means to demonstrate the utility of this novel longitudinal imaging technique. DAI causes immediate, sporadic axon damage followed by progressive secondary axon damage. We administered minocycline for three days commencing one hour after injury in one treatment group and beginning 72 hours after injury in another group to demonstrate the method’s ability to show how and when the therapeutic drug exerts protective and/or healing effects. Fewer varicosities developed in acutely treated mice while more varicosities resolved in mice with delayed treatment. For both treatments, the drug arrested development of new axonal damage by 30 days. In addition to evaluation of therapeutics for traumatic brain injury, this hybrid microlens imaging method should be useful to study other types of brain injury and neurodegeneration and cellular responses to treatment.

## Introduction

Brain injury and neurodegeneration cause multiple changes in brain cells over time, including morphology and cell loss, and can be one of several key indicators of the progression of damage and the effect of therapeutic drugs. In prior work, we developed a technique to implant a high-resolution microlens to image fluorescently labelled cells in transgenic mice before and after traumatic brain injury (TBI) and stroke^1^. In the present work, we apply this technique in a pilot study to demonstrate the utility of this technique for evaluation of drugs to mitigate damage and to optimize therapeutic windows.

An estimated 3–4 million individuals live with disabilities, including cognitive and behavioral problems, resulting from an initial TBI^2^, where diffuse TBI is the predominant subtype. Despite years of research in the field, currently no treatment is available for TBI, with the exception of palliative care^3^. Routinely, many patients who sustain a TBI either wait some time to seek medical attention or do not have access to direct treatment. Conversely, a majority of the preclinical drug studies conducted investigate dosing immediately following injury, which may partially explain poor translation in clinical studies. Furthermore, numerous pathophysiological processes occur and potentially interact after the initial injury, contributing to the current challenge of developing successful therapeutics for administration in a delayed time window. This same challenge is also seen in the translation of experimental stroke therapies into the clinic^4^. As a result, it is not only important to understand the time course of injury, but also to know how potential therapeutics intercede.

The pathophysiological response following a TBI is complex process. As a direct consequence of primary mechanical forces at the time of injury, the axonal cytoskeleton breaks down, becomes twisted and misaligned, causing the axon to become undulated^5^. Small breaks in cytoskeletal neurofilaments lead to periodic swellings along the axon known as varicosities^6–11^. In some cases, the varicosities weaken the axon, leading to the development of a terminal bulb, which retracts toward the cell body, ultimately resulting in disconnection^12–15^. The injury and repair mechanisms involve inflammatory signaling through microglia, becoming chronically activated in regions of pathology^16–18^. TBI causes changes on a cellular level, which contribute to disruption of cognitive and sensorimotor behavior, as indicated by poor neurological outcomes^19,20^. Therefore, it is necessary to monitor the progression of axonal damage, in order to identify therapies to mitigate secondary damage, including microglial activation and neurodegeneration.

Minocycline is a promising preclinical and clinical TBI therapy. In animal models, it mitigated microglia activation, owing to its anti-inflammatory properties, and it supported neuroprotection within one week following TBI^21–24^. The regulation of microglia through minocycline may contribute to the protection of axons, limiting both early and chronic neuroinflammation and possibly promoting axonal sparing^22,24–27^. Additionally, the drug can protect long-term behavioral and functional outcomes, which are negatively impacted by TBI^21,24,27,28^. While minocycline cannot prevent the early effects of mechanical damage, long term it protects white matter areas, such as the corpus callosum^27^, suggesting that the drug prevents loss or preserves axon structure.

Here, we report a novel method for longitudinal imaging before and up to 60 days after TBI using a rodent model of diffuse brain injury^1^. We also used the method to explore a new therapeutic window for minocycline. While minocycline is effective when treatment begins within 4 hr following injury^3^, it is not always possible to treat individuals in such a short timeframe. To explore expansion of the therapeutic window, we delayed minocycline treatment until 72-hr post-injury in one group of mice. This extended window represents an eighteen-fold longer therapeutic window than the 4-hr treatment. Beyond this study, the longitudinal, fluorescence imaging methods^1^ presented here should allow researchers to test the spatiotemporal effects of injury and neurodegeneration on cell morphology, connectivity, dendritic structure, and changes in neurotransmitter levels, as well as the effects of drugs to treat neuropathological conditions.

## Materials and Methods

*Animals*. Both male and female, two to four-month-old (20–30 g), C57Bl/6 J Thy1-YFPH transgenic mice (B6.Cg-Tg(Thy1-YFP)HJrs/J) were used for sham and brain injury (2–3 females per treatment group). Teklad corncob fractions (Envigo North America) were used as bedding. Mice were housed at 22 °C with a 12 h light/12 h dark cycle. Mice were provided water and food *ad libitum*. All procedures involving animals were conducted in accordance with a protocol approved by the Louisiana Tech University Institutional Animal Care and Use Committee and in accordance with the NIH Guidelines for the Care and Use of Laboratory Animals. Studies are reported following the ARRIVE (Animal Research: Reporting *In Vivo* Experiments) guidelines. Randomization of mice was achieved by assigning individuals to treatment groups prior to initiation of the study to ensure equal distribution among groups. Following surgical procedures, mice were housed individually. Data collection was stopped at pre-determined final endpoints based on days post-injury (DPI) for each animal. Mice subjected to midline fluid percussion injury had a righting time of 5–10 min. Mice were excluded from the study if net post-operative weight decreased by ≥15% of pre-surgical weight following injury (no mice were excluded). All mouse behavior was scored by investigators blinded to the treatment groups.

### Experimental design

**Figure 1:**
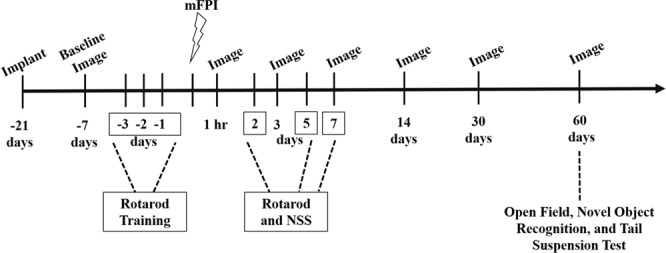
Experimental Design. GRIN lenses were implanted ≥21 days before baseline *in vivo*, two-photon imaging. Acute behavioral tests (rotarod and neurological severity score (NSS)) were conducted on days 2, 5, and 7 following midline fluid percussion injury (FPI). Mice were imaged one week prior to midline fluid percussion injury (mFPI) and at 1 hr, and at 3, 7, 14, 30, and 60 days following injury. The open field, novel object recognition, and tail suspension test were conducted on Day 60.

All mice were anesthetized (1–1.5% isoflurane) and imaged prior to injury, using a multiphoton microscope to view the brain through an implanted gradient index (GRIN) lens. The numbers and locations of axons in a three-dimensional field of view (248 μm × 248 μm × 100 μm) were recorded prior to injury to establish a baseline for each mouse. Mice were re-imaged at pre-determined time points up to 60 days (Fig. 1). Prior to the Day 30 and Day 60 time point, some mice lost their head plates, and as result, *in vivo* images were not collected beyond that time. The number of mice included at each time point can be found in Table 1. Mice with lost head plates had their craniectomies sealed with 1% low melt agarose, a cover glass, and dental acrylic. Mice with representative Day 60 *in vivo* images are available in Supplemental Results (Fig. S1). The neurological severity score (NSS) and rotarod tests were conducted on days 2, 5, and 7 post-injury to assess injury-induced sensorimotor deficits and functional recovery following brain injury and treatment, as previously described^29^. Novel object recognition, open field, and tail suspension test were performed on Day 60 to assess the long-term effect of minocycline on cognitive and affective behavior, respectively. All behavioral tests were performed in a room solely for mouse behavioral tests and analyses were performed by persons blinded to treatment conditions. Detailed methods for the behavioral tests performed at 60 days, along with the experimental results, are included in the Supplemental Data File.

**Table 1:** Number of mice imaged at each time point.

Group	1 hr	Day 3	Day 7	Day 14	Day 30	Day 60*
Sham-vehicle	4	4	4	4	3	1
TBI-vehicle	5	5	5	5	4	2
TBI-45-min mino	5	5	5	5	4	0
TBI-72-hr mino	5	5	5	5	4	3

*Image analysis not conducted at Day 60 since ≤3 mice were in each group.

Abbreviation: Minocycline, mino.

Mice were excluded from the study if herniation occurred after injury (n = 1), if injured mice did not show deficits in the rotarod or NSS tests (n = 1), if sham-vehicle mice displayed deficits following sham injury (n = 1), or if head plates were detached before Day 7 (n = 2). These mice were not included in Table 1 or in our analyses.

### GRIN lens assembly and implantation

Implantable micro-optics systems were constructed in-house by first securing a custom glass window (500-µm diameter, 600-µm length, Bern Optics, Inc., Westfield, MA, USA) to a 3-mm diameter #1.5 glass cover slip (Electron Microscopy Sciences, Hatfield, Pennsylvania, USA) with NOA 71 optically clear adhesive (Norland Products, Inc., Cranbury, New Jersey, USA). Next, a drop of the optically clear adhesive was placed on the glass window and a 500 µm diameter, 1.7 mm long, just under ½ pitch, ILH, uncoated gradient index (GRIN) lens (Go!Foton, Somerset, New Jersey, USA) was attached. Adhesive was cured for 90 min under an ultraviolet lamp. At 45 min, the imaging system was rotated 180° to ensure uniform curing. The following day, the cover slip on the lens system was secured to a stainless steel M1.6 washer (ID = 1.7 mm, OD = 4 mm, McMaster-Carr, Santa Fe Springs, CA) with optically clear adhesive and cured again for 90 min, with rotation at 45 min to complete the GRIN lens assembly.

GRIN lens assemblies were implanted, as previously described^1,30,31^. Mice were briefly anesthetized with 5% isoflurane and then injected with a ketamine/xylazine (10 mg/kg ketamine, 1 mg/kg xylazine in 0.9% saline, i.p.) cocktail. This additional anesthesia step allowed surgeons ample time to prep the mouse for surgery. Mice were transferred to a stereotaxic frame (Stoelting Co., Wood Dale, Illinois, USA) equipped with ear bars and a nose cone for isoflurane delivery (Leica Biosystems. Inc., Buffalo Grove, Illinois, USA). A SomnoSuite system (Kent Scientific, Torrington, Connecticut, USA) was used to control isoflurane delivery and a PhysioSuite system (Kent Scientific) to maintain mouse temperature at 37 °C. For the duration of the surgery, isoflurane was maintained at 1.0–1.5%. To ensure the mouse was fully anesthetized, toe pinch withdrawal was monitored every 15 min. A craniectomy was created 2.25 mm lateral and 0.9 mm rostral to bregma. A lens assembly, held on a cannula insertion tool, was implanted to a depth of 1.4 mm at a rate of 0.1 mm/min until the imaging system rested immediately superior (dorsal) to the external capsule. RelyX dental acrylate (RelyX Unicem Aplicap, 3 M ESPE, St. Paul, MN, USA) was placed around the imaging system and allowed to cure for 20 min before the mouse was returned to its home cage with a heating pad (Braintree Scientific, Inc., Braintree, Massachusetts, USA). Mice were observed until a recumbent position was maintained. To minimize post-surgical pain, an analgesic was dissolved in the mouse’s water (Children’s Ibuprophen, 30 mg/kg/day in water) for 72 hr following the procedure. Mice health and welfare were monitored for 72 hr following surgery. No intervention was necessary in any of the mice in this study. Mice were allowed to recover at least three weeks before baseline (pre-injury) imaging^1,30^.

### Head plate design and attachment

Head plates were designed in SolidWorks software and printed (MakerBot Replicator 2) in-house with polylactic acid filament. They were secured to the skull with cyanoacrylate (Super Glue, Loctite) following implantation of the GRIN lens, as previously described^31^. Head plates were designed with two cutouts. One cutout served to aid in aligning the head plate with the washer portion of the GRIN lens assembly (Supplemental Fig. S2A) The second cutout allowed for a second surgery to be performed and the injury hub^32^ attached to facilitate midline fluid percussion injury (mFPI, Supplemental Fig. S2B). The file for printing head plate and parts for the TRIO imaging support system is available at the National Institutes of Health 3D Print Exchange at https://3dprint.nih.gov/discover/3dpx-010720, Model ID 3DPX-010720^1^.

### *In vivo* multiphoton microscopy

For *in vivo* multiphoton imaging before and after mFPI, the TRIO platform was used to restrain, anesthetize, and warm the mouse for the duration of the imaging session, as previously described^31^. A Chameleon Vision-2 multiphoton laser (80 MHz, Coherent) tuned to 890 nm was used to excite YFP fluorescence and the emitted light was filtered through a 525/40 nm filter (BrightLine Filters, Semrock, Inc.). A custom, upright, multiphoton microscope system (Intelligent Imaging Innovations, Inc., Denver, CO, USA) and a 40×/0.6 NA objective with a correction collar for up to 2 mm of glass (Nikon Instruments Inc., Japan) was used to acquire scanned images^33^ of the external capsule. To acquire three dimensional (z-stack) images composed of 100 images with 1 µm step size, a dwell time of 2 µs and pixel averaging (5/scan) was used. Baseline images were acquired 3–4 weeks after lens implantation, which was one week prior to the mFPI procedure. Post-injury images were acquired at 1 hr, 3, 7, 14, 30, and 60 days after injury. Image sets were exported and stored as TIFF files on an external hard drive for offline analysis.

### Adapted mFPI surgery

The mFPI was conducted, as previously described^29,34,35^ with an adaptation made for the attachment of the injury hub. Kwik-Sil (World Precision Instruments, Sarasota, FL), a biocompatible, silicone, elastomer was used to attach the injury hub in place of dental cement to protect the GRIN lens from being removed during injury hub detachment^1^. Mice were anesthetized with 5% isoflurane and then transferred to a stereotaxic frame equipped with ear bars and a nose cone for continuous isoflurane delivery. Mice were maintained at 1.5–2.0% in air using a SomnoSuite system and a thermostatically-controlled warming pad. Isoflurane was used instead of a ketamine/xylazine cocktail, as this and other injectable anesthetics, can disrupt the injury response after TBI induction^36^. A craniotomy was created using a 3-mm trephine between lambda and bregma, along the midline without disrupting the dura. An injury hub was fabricated from a Leur lock needle hub and fixed over the craniotomy using Kwik-Sil. Injury hubs were filled with saline to check for leaks and then covered with a piece of standard lab tape to prevent bedding and other particles from accessing the exposed dura. Lab tape remained on the injury hub until it was filled with saline for injury induction. Mice were placed on a heated pad and monitored until recumbent, at which point they were returned to their housing overnight.

### Diffuse brain injury by mFPI

Diffuse brain injury by mFPI was conducted 24 hr following the attachment of the injury hub, as previously described^37^. Group sizes are indicated in the Results section. Mice were anesthetized using 5% isoflurane for 5 min. Isoflurane was used as the anesthetic, as ketamine/xylazine and other injectable anesthetics can alter the injury response following TBI^36^. The lab tape was removed from the injury hub and the intact dura was inspected for debris. The hub was filled with 0.9% sterile saline and connected to an FPI device (Custom Design and Fabrication, Virginia Commonwealth University, Richmond, VA). When mice responded to a toe pinch, an injury was administered by releasing the pendulum. Sham-injured mice followed the same protocol, except the pendulum was not released. Righting reflexes, the total time from the initial impact until the mouse righted itself, were recorded as indicators of injury. Mice subjected to mFPI had an average righting time of 426 ± 24 s, while sham-injured animals had a righting time of 23 ± 1 s. Following injury, mice were re-anesthetized with 5% isoflurane and injury hubs were carefully removed by gently pulling off the Kwik-Sil with sterilized forceps and dura was inspected for uniform herniation and integrity of the dura^35^. The craniectomy was filled with 1% low melt agarose (Amresco, Irvine, CA) and a 3 mm diameter #1.5 cover glass (Electron Microscopy Sciences, Hatfield, Pennsylvania, USA) was placed over the opening. Gel control cyanoacrylate was carefully placed around the cover glass. Next, RelyX dental acrylic was placed around and on top of the cover glass to secure the cover glass to the skull^1^. Once cured, the mouse was transferred to the TRIO platform for the 1-hr imaging time point.

### Minocycline administration protocol

Prior to injury hub attachment, mice were randomly divided into four groups as follows: sham injury with saline treatment (Sham-vehicle, n = 6), TBI with saline treatment (TBI-vehicle, n = 6), TBI with minocycline treatment beginning at 45 min post-TBI (TBI-45-min minocycline, n = 6), and TBI with minocycline treatment commencing at 72 hr post-TBI (TBI-72-hr minocycline, n = 6). Minocycline (45 mg/kg) (Tocris Bioscience) in 100 μl of 0.9% saline (Teknova, Hollister, CA), or 100 μl of vehicle, was administered intraperitoneally three times for each group. An initial dose was given at the stated time point, and then another two doses were administered at 24 hr and 48 hr after the first dose. The same mice were used for imaging and behavioral tests. The number of mice included for imaging analysis at each time point can be found in Table 1.

### Behavioral tests to assess injury

To determine if acute sensorimotor deficits were present following mFPI, rotarod performance and NSS were evaluated at Days 2, 5, and 7^29,35,38–41^. The NSS was primarily used as exclusion criterion. If injured mice had an NSS score of 3 or less, they were excluded (n = 1). If Sham-vehicle mice had a score of greater than 3, they were excluded (n = 1).

#### Rotarod

An Accurate Rotarod (AccuScan Instruments, Inc. Columbus, Ohio USA) was used to assess sensorimotor function. Mice were acclimated to the device three days before injury (acclimation phase) (Fig. 1). The mouse was placed on the stationary rod and allowed to explore the rod for 30 s. If the mouse fell off the rod before the 30 s time period, the mouse was placed back on the rod. Once the mouse had successfully explored the stationary rod for 30 s, the angular frequency of the rotarod was adjusted to a constant rate of five revolutions per min (rpm). If the mouse fell off the rod, it was placed back on the rod and the timer restarted until it could walk for 15 s. Following successful exploration, the rotarod speed was adjusted to an acceleration of 0.2 rpm/s. The trial ended once the mouse fell off the accelerating rod. The mouse was returned to its cage for 10 min and the accelerating rod portion of the test was re-run. Again, once the mouse fell, the mouse was returned to its home cage, and the acclimation phase ended. The acclimation phase was run between 12 pm and 4 pm on the day of the testing phase.

Rotarod tests were run on Days 3, 2, and 1 before injury (Fig. 1). The procedure took place at the same time each day, between 4 pm and 7 pm to minimize variability in performance related to diurnal cycles. The mice were placed on the apparatus and were tested for their ability to remain on the rotarod with an acceleration of 0.2 rpm/s. The apparatus is equipped with sensors to detect when a mouse falls and it records the latency to fall in s. Three trials were run on each testing day, with the first and second trials run one after the other and the third trial run after a 10-min break. The two longest trials were averaged for the day. The average of the two longest trials for one day before injury was used as the baseline data point. The rotarod test was conducted on Days 2, 5, and 7 following mFPI to detect injury-induced motor deficits. Since mice were not all tested on the same day, the latency to fall at Day 2, Day 5, and Day 7 was normalized to baseline latency to fall $(\frac{Day\,2,\,5\,or\,7\,Latency\,to\,Fall}{Baseline\,Latency\,to\,Fall})$ to account for both inter-animal and inter-day variability^30^.

#### Neurological severity score

To evaluate neurological impairment, which affects acute sensorimotor performance following mFPI, eight behavioral tests were performed to establish a NSS for each mouse^29,35,40,41^. The tests determined the function of the cortex and cerebellum, and if any additional motor deficits were present. The mice were assessed on the presence of (1) hind limb flexion, (2) seeking behavior, (3) startle response, ability to walk across 30 cm long, square cross section, beams with progressively smaller widths of (4) 3 cm, (5) 2 cm, and (6) 1 cm width, and to (7) balance on a 0.5 cm square beam and a (8) 0.5 cm rod with a circular cross section. Each of these 8 tasks was scored either as a one for failure or zero for success. The sum of the scores was the NSS. These tests were conducted on Days 2, 5, and 7 following injury. Data are presented as a score ranging from zero to eight, with high scores indicative of impairment.

### Image selection and parameters

Exported 16-bit TIFF files were analyzed in ImageJ (version 1.51i) software^42^. Each stack for each time point was realigned using “StackReg,” a downloadable plug-in, to correct minor misalignment of successive images caused by motion artifacts^43^. After correcting image alignment, an average intensity z projection of 11 successive images acquired in 1-µm z steps was created for each time point. An identical field of view using a particular arrangement of axons was chosen for each time point to track the same axons over time.

### Quantification of axons with undulations, varicosities and terminal bulbs

The percentage of axons with undulations, varicosities, and terminal bulbs was determined at each time point. Individuals blind to the treatment groups, compared the post-injury time points to baseline images. Observers used the drawing and region of interest (ROI) tools in ImageJ (version 1.51i) to indicate which axons had features of damage. A box was drawn around the axon and it was classified as having evidence of an undulation, varicosities, or a terminal bulb. If an axon with varicosities developed a terminal bulb, it was only counted as a terminal bulb and not both at that time point. Axons were counted if they demonstrated a feature of damage; individual features (i.e., each swelling) were not counted, since the entire axon was not in a field of view. Axons appearing normal were counted as uninjured. The ROIs were tabulated at each time point for each mouse in Microsoft Excel. Percent was calculated as follows: $\frac{Axons\,with\,feature}{Total\,axons\,in\,the\,field\,of\,view}\times 100$. Data were presented as the mean ± SD percentage of axons with each feature at each time point in each treatment group. Data were transferred to GraphPad Prism for statistical analysis^1^.

### Quantification of new varicosities at each time point

Axons with varicosities were tracked over time. At each time point, the varicosities were labeled as either newly developed or progressing from the previous time point. At each time point, the fraction of axons with newly developed varicosities was calculated by dividing the axons with new varicosities (N_t_) by the total number of axons with varicosities (V_t_). The percentage of new axons with varicosities was recorded (F_N_(t) = N_t_/V_t_*100). Data were reported as mean total percentage of axons with newly developed varicosities ± SD^1^.

### Quantification of recovered axons at each time point

Axons with varicosities were labeled as recovered if the axon returned to its baseline morphology by the subsequent time-point. For example, if an axon had varicosities at Day 3, but then recovered by Day 7, it was labeled as recovered by Day 7. The fraction of axons that recovered was calculated by dividing the number of recovered axons (R_t_) by the total number of varicosities (V_t_). The percentage of axons that recovered was recorded (F_R_(t) = R_t_/V_t_*100). Data were reported as mean total percentage of recovered axons ± SD by the respective time points^1^.

### Quantification of axon fate

To demonstrate progressive axonal injury, individual axons were tracked over time. Varicosities and terminal bulbs have been observed in healthy, adult rodents^44^. If an axon had varicosities or an axonal bulb in the baseline image, the damaged axon was excluded from future analysis (approximately two axons per mouse had varicosities or a terminal bulb in baseline images). Undulated axons at the 1-hr time point were followed over the course of the experiment and their outcome was recorded as either developing varicosities or returning to its baseline state. The percentage of undulated axons that developed varicosities or recovered back to baseline morphology was measured. Axons with varicosities were tracked over time and at the end of the 30-day time period varicosities were identified as (1) developed into a terminal bulb or lost axon, (2) recovered (no sign of injury), or (3) unrecovered (varicosities remained). The percentage of axons in the field of view that developed into a terminal bulb, recovered, or remained damaged were reported.

### Quantification of total varicosities and terminal bulbs or axon loss

The total percentage of axons with varicosities and terminal bulbs that developed over the course of 30 days was measured for each mouse. Terminal bulb and axon loss were measured together, but if a terminal bulb resulted in axon loss at the end of the experiment, it was not counted twice. Occasionally, axon loss would occur without the apparent development of a terminal bulb. The terminal bulb could have developed outside the field of view or passed through the field of view at a time point not imaged in this experiment.

### Statistical analysis

Results for each group are shown as the mean ± SD^45^. Repeated measure analysis of variance (ANOVA) was performed to analyze the behavioral tests as the same subsets/cohorts of mice were analyzed over time. To analyze the NSS data, we used the Scheirer–Ray–Hare test since NSS are ordinal variables and do not follow normality assumption, necessary in ANOVA. Scheirer–Ray–Hare test is a nonparametric procedure to test significant differences among groups of independent variables on a continuous or ordinal dependent variable. ANOVA was used to test if there was a significant difference in the amount of damage (measured in the percentages of axons undulated, developed to varicosities, and TD) among the four groups. Power analyses for the ANOVA tests were performed using eta squared as the effect size (${\eta }^{2}=\frac{{{\rm{SS}}}_{{\rm{trt}}}-{{\rm{SS}}}_{{\rm{res}}}}{{{\rm{SS}}}_{{\rm{trt}}}}$, where *SS*_*trt*_ and *SS*_*res*_ represent the sum of squares for treatment and residuals, respectively). A Bonferroni correction for multiple comparisons was used for pairwise comparisons in behavioral test analyses. Pairwise Wilcoxon rank sum test was used for pairwise comparison in NSS data analyses since the scores are ordinal. Tukey’s test was used to perform pairwise comparisons in brain damage analyses. For the power analysis of pairwise comparison tests we used Cohen’s d effect size (${\rm{d}}=\frac{{\bar{X}}_{1}-{\bar{X}}_{2}}{\sqrt{{s}_{p}^{2}}}$, where ${s}_{p}^{2}$ measures the pooled sample variance). All tests were performed using R packages at *α*  =  0.05 level of significance. The *p-values* and F statistics are reported individually in the figure legends or results section, along with the statistical analysis used for the data set. The number of mice included in the imaging analysis at each time point is listed in Table 1.

## Results

### Righting reflex as an indicator of brain injury severity

Mice were either subjected to moderate mFPI diffuse brain injury (n = 15) or sham injury (n = 4), in which the mice undergo the same procedures as the brain-injured group except the pendulum on the device was not allowed to strike the fluid percussion piston.

**Figure 2:**
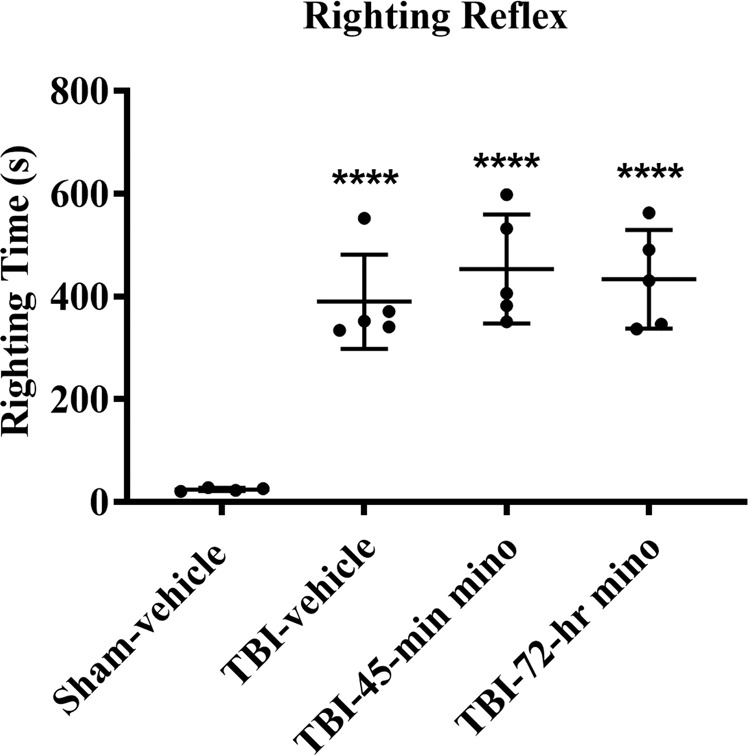
Mean righting reflex times. Traumatic brain injured (TBI) mice sustained a midline fluid percussion injury and had significantly longer righting reflex times than Sham-vehicle mice. ****p < 0.0001 vs. Sham-vehicle. ANOVA with Bonfererroni t-test for pairwise comparisons (p < 0.0001, ANOVA power = 1.00). Data presented as mean ± SD.

Figure 2 represents the mean righting time in each experimental group (Sham-vehicle, n = 4; TBI-vehicle, n = 5; TBI-45-min minocycline, n = 5; TBI-72-hr minocycline, n = 5). All brain-injured groups had significantly longer righting times than the Sham-vehicle mice.

### Minocycline reduced acute sensorimotor impairment post-injury

**Figure 3:**
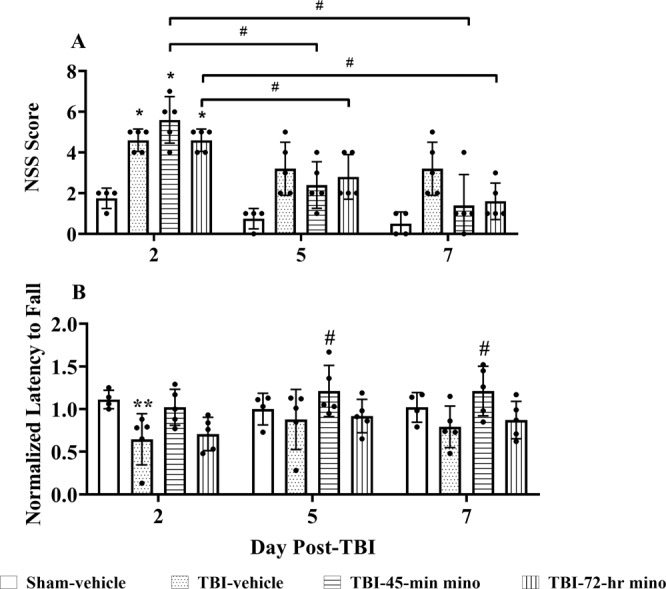
Minocycline reduced acute sensorimotor impairment. (**A**) NSS results. Minocycline (mino in legend) reduced sensorimotor impairment over one-week post-TBI. Scheirer–Ray–Hare test results show both treatment groups and time are significant factors (p = 0.0004 for treatment groups and p = 0.00004 for time). Pairwise Wilcoxon rank sum tests were used for pairwise comparison among groups at each time point. The TBI-vehicle mice had significant differences as compared to the Sham-vehicle mice on Day 2 (D2, p = 0.035); no significant differences existed on Day 5 (D5) or Day 7 (D7). The TBI-45-min minocycline treated mice had significant deficits versus Sham-vehicle treated mice only on Day 2 (p = 0.035); in contrast, there was no significant difference on D5 (p = 0.08) and 7 (p = 0.41) between these two groups. TBI-72-hr minocycline treated mice had significant deficits on Day 2 (p = 0.035) versus Sham-vehicle and no significant differences at D5 and D7 (0.09 and 0.12, respectively). Between test days, TBI-45-min minocycline treatment scores decreased significantly from D2 – D5 (p = 0.023) and D2–D7 (p = 0.023), but not D5 – D7 (p = 0.19). Similarly, the TBI-72-hr group scores decreased significantly from D2 – D5 (p = 0.04) and D2 – D7 (p = 0.03), but not D5 – D7 (p = 0.10). Data are presented as mean ± SD *p < 0.05 vs. Sham-vehicle, ^#^p < 0.05 between days for same treatment group. (**B**) Rotarod test results. Results were normalized to baseline latency to fall (Lee *et al*. 2016). TBI-vehicle mice performed more poorly than Sham-vehicle mice on Day 2 (p = 0.007). TBI-45-min minocycline (mino in legend) treated mice performed significantly better than the TBI-vehicle treated mice on Day 5 and Day 7 (p = 0.04 each). The TBI-72-hr minocylcine treated mice did not perform significantly better than TBI-vehicle mice at any time point (p > 0.05), nor was the performance significantly worse than TBI-45-min minocycline-treated mice (p > 0.05). Data are presented as mean ± SD. *p < 0.05, **p < 0.01 vs. Sham-vehicle, #p < 0.05 vs. TBI-vehicle. Sham-vehicle (n = 4), TBI-vehicle (n = 5), TBI-45-min minocycline (n = 5), TBI-72-hr minocycline (n = 5), repeated measures ANOVA followed by Bonferroni correction (Group: p = 0.05, F = 3.303 Time: p = 0.03, F = 3.320, Group/Time interaction: p = 0.03, ANOVA power = 0.06).

The NSS was conducted during the first week post-injury (Fig. 3A). On Day 2, the TBI-vehicle, TBI-45-min, and TBI-72-hr minocycline treated mice had significant deficits as compared to the Sham-vehicle mice, as expected. Both groups of minocycline treated mice had significantly improved NNS scores from Day 2 to Day 5 and Day 2 to Day 7. However, there was no significant difference in mean scores between Day 5 and Day 7 for either group.

### Minocycline reduced motor deficits over one-week post-injury

The accelerating rotarod test was conducted on Day 2, 5, and 7 to assess motor deficits (Fig. 3B). The latency to fall on Day 2, 5, and 7 was normalized to baseline latency to fall to account for inter-animal variability. On Day 2 post-injury, the TBI-vehicle mice had significant motor deficits as compared to the Sham-vehicle mice. On Day 5 and 7, TBI-45-min minocycline treated mice performed significantly better than TBI-vehicle mice. The TBI-72-hr minocycline treated mice did not perform significantly different from TBI-vehicle or TBI-45-min minocycline mice at any time-point. The administration of minocycline commencing at 45 min following injury reduced motor deficits over 1 week following mFPI as compared to the TBI-vehicle treated mice.

### Time-course images of axonal damage following mFPI and minocycline administration

**Figure 4:**
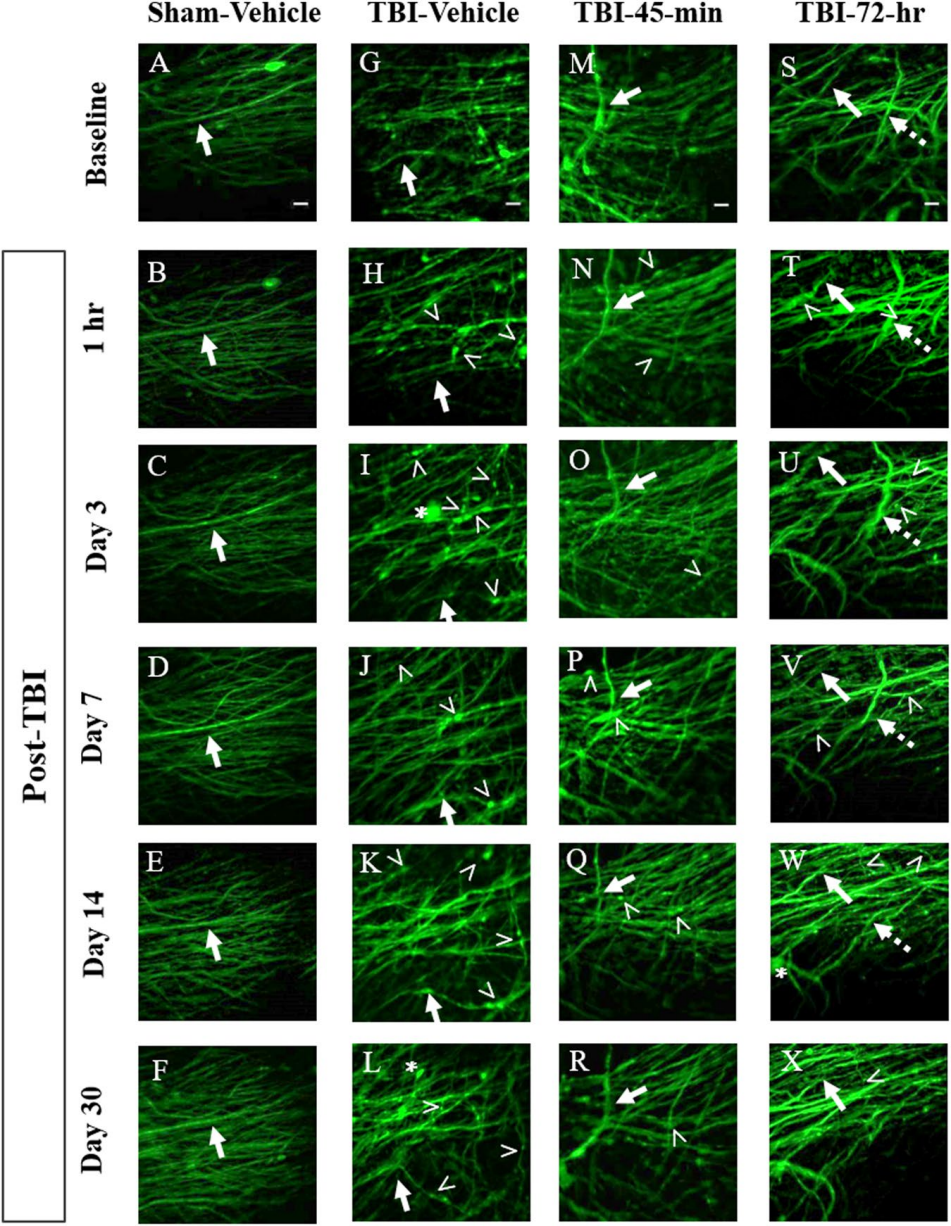
Time course images of axonal damage and repair following midline fluid percussion injury (mFPI) and minocycline treatment. (**A–F**) Sham-vehicle mice have uniform axon morphology over the course of 30 days. In the injured groups, TBI-vehicle (**G–L**), TBI-45-min minocycline treated (**M–R**), and TBI-72-hr minocycline treated (**S–X**) mice all exhibit hallmarks of diffuse axonal injury (DAI) including varicosities (>), terminal bulbs (asterisks), and disappearing axons (dashed arrow). These hallmarks were quantified over the course of 30 days to demonstrate the effect of treatment on secondary axonal damage and repair. For each mouse, axons forming distinctive patterns in baseline images were used as landmarks. These axons were followed in each mouse throughout the time series to ensure that the same group of axons were imaged over time. One such axon is identified by a solid arrow in each panel. Images are z-stack projections (5 planes, 1 µm apart) of the same field of axons over time. Scale bars are 10 µm.

Images of the same brain region in live mice were collected over the course of 30 days to determine the time course of axonal damage following mFPI in both sham-injured (Fig. 4A–F) and brain-injured, untreated (Fig. 4G–L) mice. Images were also acquired to monitor the effect of minocycline on secondary axonal damage at both a known therapeutic time window (TBI-45-min mino, Fig. 4M–R) and at a novel, expanded therapeutic window (TBI-72-hr mino, 4S-X). In brain-injured mice, varicosities (Fig. 4,>), terminal bulbs (Fig. 4, asterisk), and disappearing axons (Fig. 4, dashed arrow) were observed. These hallmarks of DAI^46^ were quantified over the course of 30 days to demonstrate the effect of minocycline versus saline treatment on the progression of axonal injury and repair.

### Minocycline does not protect against mechanical damage

**Figure 5:**
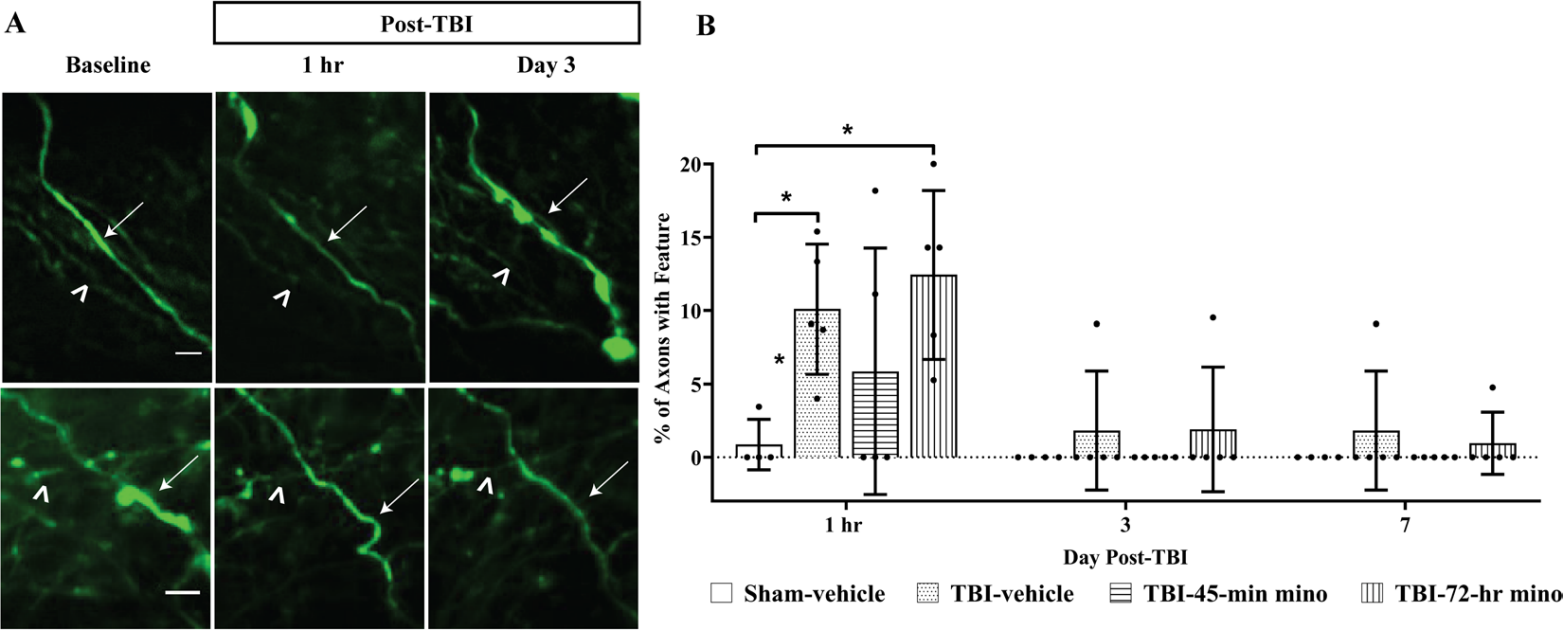
Mechanical axon damage following midline fluid percussion injury (mFPI) and minocycline treatment. (**A**) Representative image of axons with injury-induced undulated features. Undulated axons either developed varicosities (top panel, arrow) and/or terminal bulb (top panel, asterisk) or return to baseline morphology (bottom panel). Identical axons throughout imaging sessions are marked with an arrowhead. Scale bar for top panel = 5 µm, bottom panel = 10 µm (**B**) Undulated axons were apparent at the one-hr time point, independent of treatment. Two-way ANOVA results show that both treatment groups (p = 0.005) and time (p < 0.001) are significant factors. Specifically, at one hour, there is a significant difference between TBI-7-hr and Sham-vehicle (p = 0.012) and between TBI-Vehicle and Sham-vehicle (p = 0.050), Tukey test. Some undulated axons persisted until Day 7, but by Day 14 and Day 30 undulated axons were no longer observed. Data are presented as mean ± SD. Scale bar = 10 µm.

**Figure 6:**
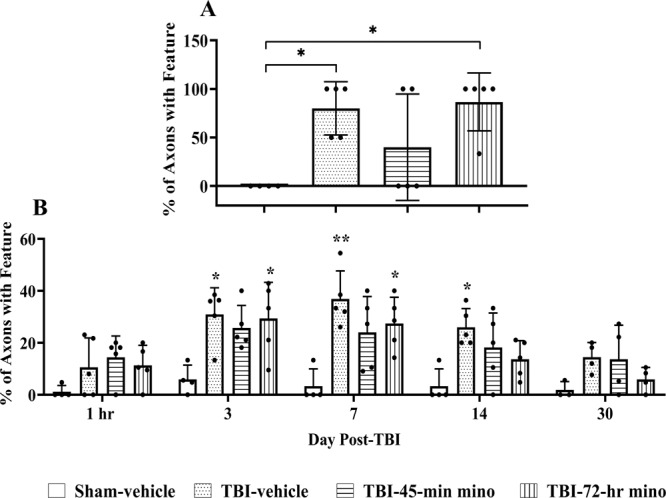
Development of undulations and varicosities. (**A**) Effect of minocycline on undulations developing into varicosities. Measured over 30 days. Significant differences were observed in the percentage of undulations that developed to varicosities between the Sham-vehicle and TBI-vehicle (p = 0.02) groups, and TBI-72-hr-minocycline and Sham-vehicle groups (p = 0.01). Data are presented as mean ± SD. Number of mice imaged is listed in Table 1. *p < 0.05 vs. Sham-vehicle, one-way ANOVA was performed to test the percentage of undulations that developed to varicosities (p = 0.008, ANOVA power = 0.96). Tukey’s test was performed for pairwise comparisons. (**B**) Axons with varicosities at 5 time points after experimental TBI. The percentage of axons with varicosities was measured at each time point. Two-way ANOVA shows that main effects of treatment (F=18.82, p < 0.00001) and time (F = 10.64, p < 0.00001) are significant. Tukey’s test was performed for pairwise comparisons. The Tukey test results show a significant difference between TBI-vehicle (p < 0.0001), TBI-45-min minocycline treated mice (p < 0.0001), TBI-72-hr minocycline treated mice (p < 0.0001) as compared with Sham-vehicle mice. All TBI groups exhibited axons with varicosities, independent of treatment. TBI-vehicle treated mice had significantly more axons with varicosities on Day 3 (p = 0.011), Day 7 (p = 0.002), and Day 14 (p = 0.01) compared to the Sham-vehicle mice. The percentage of axons with varicosities in TBI-45-min minocycline treated mice (mino in legend) were not significantly different than the Sham-vehicle mice at any time point. The TBI-45-min minocycline treated mice had a non-significant, smaller percentage of axons with varicosities than the TBI-vehicle mice. The TBI-72-hr minocycline treated mice had significantly more axons with varicosities on Day 3 (p = 0.018) and Day 7 (p = 0.029) as compared to the Sham-vehicle mice. By Day 14, the TBI-72-hr minocycline treated mice had a non-significant, smaller percentage of axons with varicosities than the TBI-vehicle treated animals (p = 0.188). Data are presented as mean ± SD. *p < 0.05, **p < 0.01.

Undulated axons were identified as having a rippled profile (Fig. 5A, arrows)^11,12^ and represent mechanical damage from the diffuse brain injury forces. Undulations were apparent at 1-hr post-injury. A few undulations persisted on Days 3 and 7, but by Day 14, no axons with undulated features were within the field of view (Fig. 5B). Minocycline had no effect on the development of undulations, as expected, since undulations are a direct result of mechanical forces applied during the induction of injury. Axons with undulations were tracked over time to determine if undulated axons from primary damage developed into varicosities (Fig. 5A, top panel) or returned to the baseline morphology (Fig. 5A, bottom panel). In both the TBI-vehicle and TBI-72-hr minocycline treated group, significantly more of the observed undulated axons developed into varicosities as compared to the Sham-vehicle mice. While the TBI-45-min minocycline treated mice had more observed undulations develop into varicosities as compared to the Sham-vehicle mice, it was not significant (Fig. 6A).

### Development of varicosities and terminal bulbs following mFPI and minocycline administration

Axons with varicosities were identified by the presence of periodic swellings, as compared to their baseline. At each time point, the percentage of axons in the field of view with varicosities was measured (Fig. 6B). At one hr, all brain-injured mice developed varicosities, independent of treatment. TBI-vehicle treated mice had a significantly higher percentage of axons with varicosities than the Sham-vehicle mice at Days 3, 7, and 14. The TBI-45-min minocycline treated mice were not significantly different from the Sham-vehicle mice at any time point. On Day 14, the TBI-45-min minocycline treated mice had a smaller, nonsignificant percentage of axons with varicosities as compared to the TBI-vehicle mice. The TBI-72-hr minocycline treated group had significantly more varicosities at Day 3 and Day 7 as compared to the Sham-vehicle mice. However, by Day 14, the TBI-72-hr minocycline treated mice had fewer varicosities as compared to the TBI-vehicle treated mice, although significance was not achieved. At Day 30, there was no significant difference between any group. Some single swellings appeared in uninjured brains that disappeared over time. Isolated swellings (i.e., not periodic) occur occasionally in adult rodents and are not associated with injury^44^; thus, these were not included.

**Figure 7:**
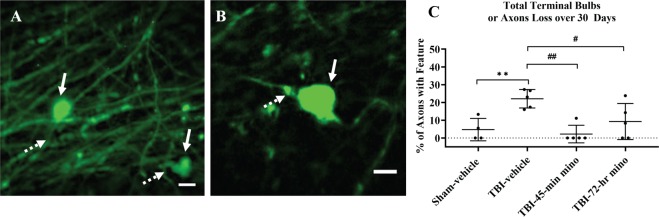
Terminal bulbs. (**A,B**) Terminal bulbs are identified as swellings (solid arrow) at the distal end of axons (dashed arrow). Images are from different mice and are cropped to show injury features. Scale bar = 10 µm. **(C)** Tukey’s test for pairwise comparison shows a significant difference between TBI-vehicle and Sham-vehicle treated mice (p = 0.01), TBI-vehicle and TBI-45-min minocycline mice (p = 0.002), and TBI-vehicle and TBI-72-hr minocycline mice (p = 0.049). Data are presented as mean ± SD. **p < 0.01 vs. Sham-vehicle; ^#^p < 0.05, ^##^p < 0.01 vs. TBI-vehicle, one-way ANOVA (p = 0.002, ANOVA Power = 0.99), numbers of mice are listed in Table 1.

Terminal bulbs were identified as large swellings (Fig. 7A,B, dashed arrow) at the end of axons (dashed arrows). Over the 30-day time period, the TBI-vehicle treated mice developed significantly more terminal bulbs than the Sham-vehicle mice (Fig. 7C). Both minocycline treated groups had a significantly smaller percentage of axons with terminal bulbs or axon loss than the TBI-vehicle treated group. Minocycline mitigated further axonal damage and prevented axon loss.

### Evolution of secondary damage is reduced with minocycline treatment at 30 day time point

**Figure 8:**
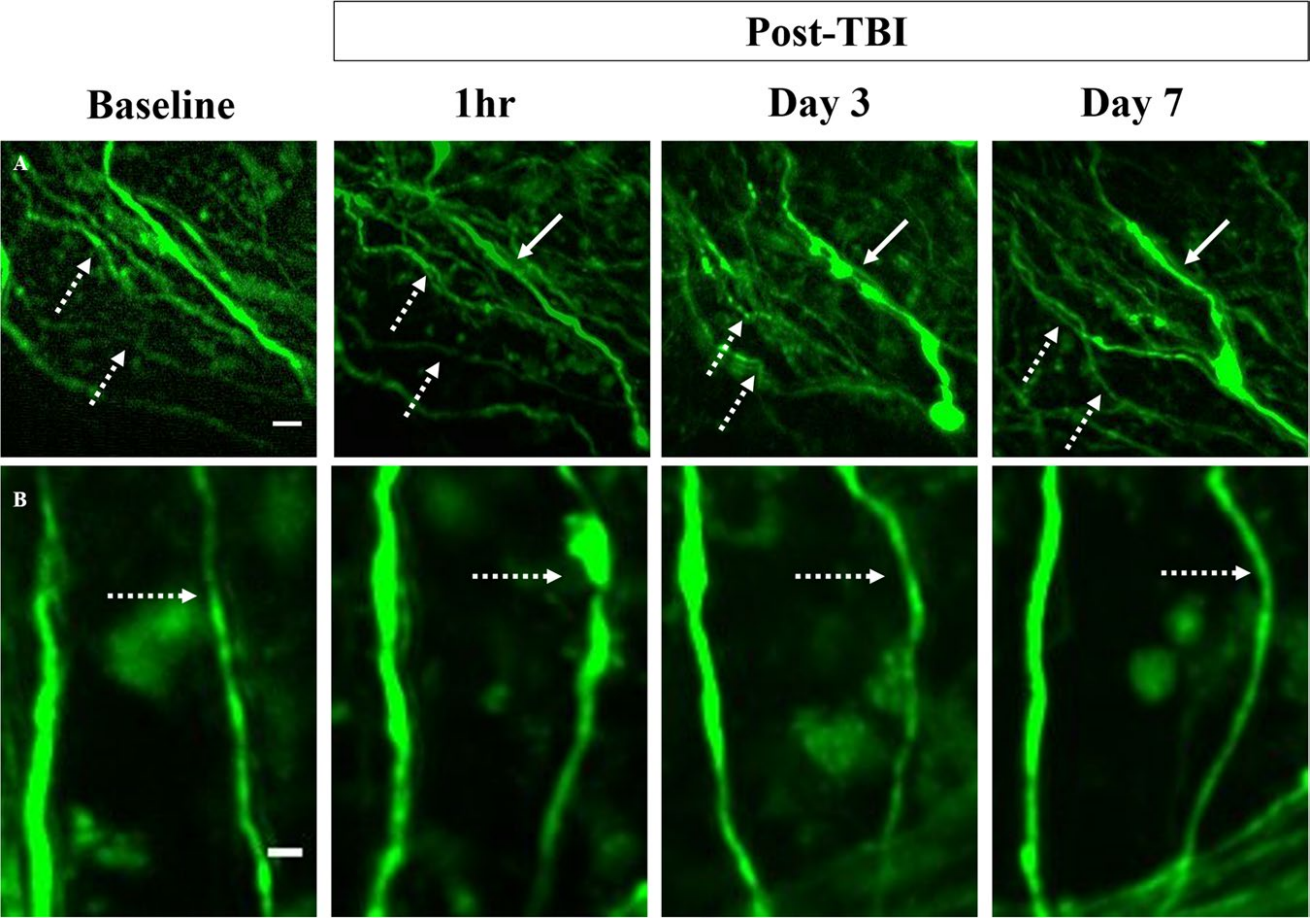
Varicosities tracked over time. (**A,B**) Axons with varicosities were tracked over time to determine if they persisted (solid arrow) or recovered and returned to their baseline morphology (dashed arrow). Images are from two different mice treated with minocycline at (**A**) 45-min and (**B**) 72-hr. Scale bar = 10 µm in **A**, scale bar = 5 µm in **B**.

Axons were tracked over 30 days to determine if minocycline treatment influenced axonal recovery or secondary injuries, such as delayed varicosity development or terminal bulb formation. Axons with varicosities were classified as either recovered (Fig. 8, dashed arrow), persisting (Fig. 8, solid arrow), or developing into a terminal bulb at the end of the 30-day time period. One mouse in each group lost a head plate before Day 30 (Table 1) which prevented further imaging. As expected, the Sham-vehicle treated group had a significantly smaller percentage of axons with varicosities^44^ develop over the 30-day time period as compared to TBI-vehicle treated mice (p = 0.002) (Table 2). Of the axons with varicosities (54.7% ± 9.1%) in the TBI-vehicle group, 47.5% ± 14.3% recovered, 26.6% ± 8.4% remained unresolved, and 25.9 ± 13.7% of these axons developed terminal bulbs or resulted in axon loss. The TBI-45-min minocycline mice had a non-significant higher percentage of axons with varicosities than the sham-vehicle (p = 0.10) over the 30-day time period, and this group had a non-significant smaller percentage than the TBI-vehicle mice (p = 0.08; Table 2). Notably, none of the tracked axons with varicosities in the TBI-45-min minocycline treated group developed terminal bulbs or axon loss. Occasionally, terminal bulbs and axon loss were identified without observing varicosities at a prior time point (Fig. 4, dashed arrow). The occurrence of terminal blubs and axon loss in this population of axons was significantly less than the TBI-vehicle group (p = 0.002, p = 0.049, for TBI-45-min and TBI-72-hr, respectively, (Fig. 7). Because the injury is diffuse, some axons could have developed varicosities outside of the field of view or between imaging time points. However, while none of the tracked axons developed terminal bulbs or axon loss in the TBI-45-min minocycline treated mice, about half of the tracked axons with varicosities persisted (45.8% ± 45.9%). The TBI-72-hr minocycline mice had a non-significant, higher percentage of axons with varicosities develop over the 30 days than the sham-vehicle mice (p = 0.08) (Table 2). None of the tracked axons developed into terminal bulbs and the majority of the damaged axons recovered (78.3% ± 15.6%) back to their baseline state by 30-days. Administration of minocycline commencing at 45 min resulted in the lowest percentage of varicosity development (33.1 ± 5.3%) over the course of 30 days after injury. However, the data suggest that there was little therapeutic effect of acutely-administered minocycline on repairing axonal damage. In contrast, administration commencing at 72 hr did not protect against early development of varicosities, as would be expected with delayed administration. However, the majority of the damaged axons eventually returned to the baseline state, suggesting that later administration of minocycline promoted recovery.

**Table 2:** Fate of axons with varicosities over 30 days.

Treatment group	Axons with varicosities (%)^a^	Axons with varicosities returning to baseline (%)^b^	Axons with varicosities persisting until Day 30 (%)^c^	Developed terminal bulbs or resulted in axon loss (%)^d^
Sham-vehicle	5.3 ± 5.6	75.0 ± 35.3	16.7 ± 28.9	0.0 ± 0.0
TBI-vehicle	54.7 ± 10.4**	47.5 ± 14.3	26.6 ± 8.4	25.9 ± 13.7*
TBI-45-min mino	33.1 ± 5.3	54.2 ± 45.9	45.8 ± 45.9	0.0 ± 0.0 ##
TBI-72-hr mino	40.5 ± 14.2	78.3 ± 15.6	21.7 ± 15.8	0.0 ± 0.0 #

Data are presented as mean ± SD. Only mice with head plates attached until Day 30 were included in the analysis (Table 1). ^a^Percentage of total axons in the field of view with varicosities measured over 30 days, both forming early and late (p = 0.004, ANOVA power = 0.98, Tukey’s test for pairwise comparisons). Last three columns are subsets of the first data column. ^b^Percentage of axons with varicosities identified at 1 hr, Day 3, Day 7, and Day 14 that recovered by Day 30 (p = 0.46, ANOVA power = 0.39). ^c^Percentage of axons with varicosities persisting until Day 30 (p =0 0.58, ANOVA power = 0.30). ^d^Percentage of axons with varicosities that developed terminal bulbs or resulted in axon loss (p = 0.0066, ANOVA power = 0.99, Tukey’s test for pairwise comparisons). *p < 0.05, **p < 0.001, vs. Sham-vehicle, ^#^p < 0.05, ^##^p < 0.01, vs. TBI-vehicle, varicosities initially, axons returning to baseline, persisting varicosities, varicosities to terminal bulbs or axon loss. One-way ANOVA and Tukey’s test for pairwise comparisons. Abbreviation: minocycline, mino.

**Figure 9:**
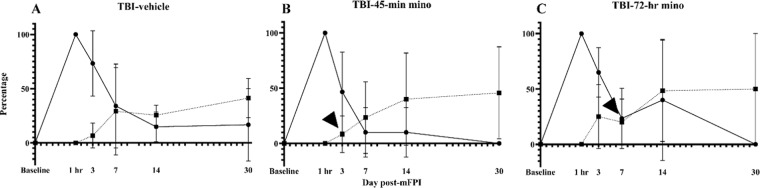
Minocycline mitigates if not arrests secondary injury and allows damaged axons to recover. The percentage of newly injured and recovered axons was calculated at each time point by dividing the number of new varicosities (N_t_) and recovered varicosities (R_t_) by the number of varicosities (V_t_) at each time point, (F_N_(t) = N_t_/V_t_*100, F_R_(t) = R_t_/V_t_*100). At the 1-hr time point, all varicosities were new; thus, the values are 100% for all groups (solid lines, circles). Likewise, no axons were classified as recovered at 1 hr; these values are 0% for all groups (dotted lines, squares). (**A**) The TBI-vehicle group had a percentage of new axons with varicosities that exceeded 20% at each time point, with little recovery. At Day 30, the TBI-vehicle group continued to show features of evolving secondary damage, as evidenced by 16.7% percent of new varicosity development. In the TBI-45-min minocycline (**B**) and TBI-72-hr minocycline (**C**) groups, a higher percentage of axons had recovered by Day 7 and Day 14, respectively. This recovery surpassed the percentage of new axons with varicosities and occurred a week after the end of the treatment period for each (arrowhead), suggesting long-lasting effects on both secondary injury and recovery. At Day 30, both minocycline treatment groups had no new varicosities. Two-way ANOVA (Percentage of new varicosities, Time: p = 0.001, Treatment p = 0.09; Percentage of recovered varicosities, Treatment: p = 0.73, Time: p = 0.01). Data presented as mean ± SD.

We conducted an additional assessment to determine when a treatment condition was contributing to the recovery or preventing further damage. We determined the percentage of new axons with varicosities and the percentage of recovered axons at each time point (Fig. 9). At Day 3 post-mFPI, all groups had a higher percentage of new damage occurring than recovery, as seen by the higher percentages of axons with new varicosities (solid line, circle) than the percentage of recovered axons (dashed line, square). As expected, new damage was lower in the TBI-45-min treatment group compared to vehicle and TBI-72-hr group at the Day 3 time point. New varicosity development continued to decrease by Day 7 for all groups with the TBI-vehicle-treated group maintaining a higher percentage versus TBI-45-min minocycline. New varicosities continued to develop in TBI-vehicle-treated mice at each remaining time point, suggesting persisting secondary damage^12^. At Day 30, 16.7 ± 33.33% of the axons had newly formed varicosities, demonstrating continued secondary injury in TBI-vehicle mice. In contrast, no new damage developed in tracked axons in either minocycline-treated groups by Day 30 (Fig. 9B,C). Recovery occurred in each minocycline treatment group with a relatively low percentage of axons with varicosities that had resolved by Day 3 and a larger percentage by Day 7. Recovery and new damage were equivalent and unchanged from Day 7 to Day 30 after brain injury in untreated mice. After completion of therapy (Fig. 9B,C, arrowhead), the percentage of recovered axons continued to increase, although this was not statistically significant when compared across all time points. These results suggest that minocycline not only attenuates and eventually arrests new secondary damage, but it also promotes axonal recovery.

### Behavioral tests conducted at Day 60 post-injury

At Day 60, three behavioral tests were conducted to demonstrate that neurobehavioral tests can be conducted on the same mice that are used for imaging to assess the neurological effect of minocycline on long-term behavioral outcomes. In both the tail suspension test (Fig. S3) and open field (Fig. S4), no difference was found between treatment groups. However, at Day 60, TBI-45-min minocycline treated mice spent significantly more time exploring the novel object rather than the familiar object during the novel object recognition test (p = 0.04) (Fig. S5A). Both the TBI-vehicle and TBI-72-hr treated mice spent similar amounts of time with the familiar and novel object. However, no significant difference was found between the discrimination index for each of the four treatment groups (Fig. S5B). These data represent group sizes that were powered for imaging outcomes; thus, the behavioral data must be interpreted with caution.

## Discussion

In this pilot study, we demonstrated the utility of our novel longitudinal imaging method^1^ for preclinical drug testing that allows researchers to observe the timing and magnitude of the effects of drugs at high cellular resolution for a variety of neurodegenerative diseases. This study extends the mechanistic understanding of DAI as axonal pathologies develop and resolve in subcortical white matter. By combining transgenic reporter mice and GRIN lens two-photon imaging, we have advanced the understanding of axonal injury, degeneration, and recovery in the diffuse injured brain, as well as refined the time course of protection of minocycline at a cellular level. At two weeks post-injury, delayed minocycline administration had the same net neuroprotective effects against secondary damage as acute administration, although with seemingly different rates of secondary injury and recovery. Both acute and delayed treatment appeared to arrest development of new damage by 30 DPI, which was in sharp contrast to continued development of new damage in TBI-vehicle treated mice.

To date, research pertaining to the progression of axonal damage and degeneration after TBI has been conducted with *in vitro* techniques and traditional histological approaches in animal studies^7,8,11,12,16,46–48^. While these methods have offered insights into DAI and secondary injury, individual axons cannot be followed over time to observe the natural and drug-induced recovery of axonal damage that is a confounding factor in comparing the progression of axonal damage over time. Unlike previous studies, we followed individual axons a week before and for over 30 days post-TBI to determine the time course and fate of each injured axon, including the time course of the effect that minocycline had on the reduction of secondary damage and the recovery of axonal damage.

We successfully followed the same axons in intact mouse brain, before and after TBI and confirmed key observations from earlier *in vitro* and histological studies. For example, we observed that the mechanical forces from the initial injury lead to undulated axons, which quickly develop varicosities in less than one hr. In fact, by 1-hr post-injury there were few undulated axons, and axons had either recovered or developed varicosities in all injured groups. This aligns with prior studies, as axons of cultured cells subjected to stretch injury relaxed or became permeable by 45-min post-stretch^5^ and axons in an animal model of FPI developed varicosities in as little as 15 min post-injury^12^. Once axons become damaged, they are susceptible to disconnection, leading to the development of terminal bulbs and ultimately, axon loss^12,46,49^, which we also observed. Furthermore, we also showed that damaged axons do not always develop terminal bulbs but are capable of recovering back to their baseline morphology^50,51^. We further demonstrated that recovery can happen rapidly or slowly, over several days, and that it occurs more frequently with minocycline treatment. Additionally, a recent study using CLARITY demonstrated that varicosity size decreases with survival time, alluding to axonal recovery mechanisms that reinstate transport^52^. Excitingly, our longitudinal study has allowed us to observe the reduction in size of individual axonal swellings over time, directly supporting this finding. The temporal resolution of this technique has not only allowed us to observe spontaneous recovery of axons following injury, but has also demonstrated the efficacy of minocycline, a potential treatment, at the individual axon level.

Our longitudinal imaging approach was further validated by confirming that when minocycline is administered at 45 min after injury, it protects the white matter over time, as others have shown^22,23,53^. In addition, this system was used to determine the effectiveness of expanding the therapeutic window of minocycline treatment to 72 hr after injury. While administration at this late time point did not protect against overall varicosity development, it also protected white matter long term. Furthermore, it appeared to result in a higher percentage of recovered axons. Some differences between treatment groups at a particular time point were often not significant. Our study was powered for repeated measurements over the duration of the study. Prospective preclinical studies would require additional mice per condition to confirm drug effects at specific time points or sex-dependent differences.

Minocycline has long-term protective benefits following TBI when administered up to 4 hr post-injury^22–28,53,54^. At 24 hr post-injury, minocycline significantly reduced the number of CD11b + cells, a marker for inflammation. At 3 months following experimental closed head injury, minocycline significantly protected the volume of the corpus callosum^27^. These studies suggest, while minocycline does not have immediate protective benefits, it protects white matter long term. However, prior studies did not determine exactly how and when minocycline exhibits its protective benefits.

Minocycline, regardless of the dosing strategy, did not protect against the development of varicosities from initial mechanical damage in the present study, as others have reported^22^. In both treatment groups, a similar percentage of undulated axons at the 1-hr time point developed varicosities, compared to the saline treated mice (Fig. 6). This is not a surprise, as both treatments were administered in a time window after application of mechanical force to the brain that initiates undulations, cytoskeletal damage, and vesicle transport disruption in injured axons. Therefore, post-injury treatment could not prevent this damage, which has been shown to occur within 15 min post-mFPI^12^. Future studies that explore resilience or priming strategies could have an impact on primary mechanical injury to axons. Yet, minocycline treatment had significant therapeutic efficacy in our study with mitigation of secondary injuries, such as varicosities and terminal bulbs, which can otherwise lead to axon loss. One limitation of our study was that the percentage of undulated axons was low with only 2–3 undulated axons in any injured group (Fig. 5B). This could be due to the timing of our first image acquisition, which was one hr after injury. The small breaks resulting from undulations cause a disruption in axonal transport, leading to varicosities in as little as 15 min^12^. For future studies, adding a time window within 15–30 min immediately following injury may reveal a higher number of undulated axons that can be better tracked over time.

Distinct protective profiles emerged between early and delayed treatment in our study. Administration from 45 min through 3 DPI significantly reduced overall damage, whereas delayed administration appeared to establish a more permissive environment for axonal recovery (Table 2). Possibly the well-known, potent anti-inflammatory mechanism of minocycline is better utilized for recovery when delayed^25,55–58^. However, chronic delayed dosing may interfere with microglial activation necessary for repair, as suggested by a clinical study in adults who had a TBI at least 6 months prior to a 12-week treatment with minocycline. Some patients had a biomarker of axonal degeneration, which could have resulted from decreased microglial activation^59^. Perhaps an acute and then slightly delayed, subacute treatment scheme may obviate the need for chronic dosing or enable smaller chronic doses of the drug over time to maintain protective benefits against secondary damage without risking neurodegeneration.

The ability to image the same cells before and following TBI, or development of other types of neurodegeneration, minimizes inter-animal variability, and thus reduces the number of animals required for studies. We had excellent statistical power for most of our end-point comparisons, except for the behavioral tests, which had high variability. Male and female mice were included in each treatment group resulting in low numbers per sex. This may have contributed to the large variance and non-significant differences in neurological function at 2 months post-injury, since female rodents may outperform males in cognitive function and motor coordination tests^60–62^. However, all injured animals exhibited acute neurological impairments that resulted in acute sensorimotor deficits, providing an experimental TBI that models clinical symptoms of diffuse brain injury. The injury-induced neurological symptoms were unaffected by the earlier lens surgery or the presence of the head-stage assembly, as we have previously demonstrated^30^.

Findings from this study have broadened our understanding of the effects of DAI and of minocycline on protection and recovery up to 30 days after traumatic brain injury. However, this was a proof-of-concept project intended to demonstrate the capabilities of our imaging method and was not a full-scale preclinical trial or a comprehensive basic research study. As such, some of the results within a particular image day lack sufficient statistical power (most comparisons of cumulative data over 30 days were adequately powered). To confirm trends seen between treatments at a specific time point, future experiments would require more mice per group. Future work would also include evaluation of chemical biomarkers, additional types of behavioral tests, functional imaging, with genetically-encoded fluorescent reporters, such as iGluSnFR to monitor neurochemical signal dynamics. We also anesthetized our mice in this study, which was not a problem for this study of cell-level damage. However, when using fluorescent reporters to record neurochemical signals, anesthesia should not be used. In this case, mice can be head-fixed in a device such as the Mobile Home Cage (Neurotar) or a trackball, both of which facilitate behavioral tests during image acquisition.

In summary, we have developed an *in vivo*, high-resolution, optical imaging technique to track individual cells deep in the mouse brain to monitor changes in the same cells over several weeks. Using this technique in a brain injury model, we identified the fate of individual axons shortly after brain injury through a time that included the development of secondary damage and we found that delayed dosing of minocycline may enhance recovery. Our technique is applicable to brain disorders involving cell loss and changes in cell morphology, connectivity, dendritic structure, and changes in neurotransmitter levels. This method should be useful for studies ranging from preclinical trials to basic neuroscience research in areas such as brain injury, aging, addiction, and neurodegeneration.

## Supplementary information

Supplementary Information.

## References

[1] Pernici CD, Kemp BS, Murray TA (2019). Time course images of cellular injury and recovery in murine brain with high-resolution GRIN lens system. Sci. Rep..

[2] Zaloshnja E, Miller T, Langlois JA, Selassie AW (2008). Prevalence of long-term disability from traumatic brain injury in the civilian population of the United States, 2005. J. Head. Trauma. Rehabil..

[3] Diaz-Arrastia R (2014). Pharmacotherapy of traumatic brain injury: state of the science and the road forward: report of the department of defense neurotrauma pharmacology workgroup. J. Neurotrauma.

[4] Cui LL, Golubczyk D, Tolppanen AM, Boltze J, Jolkkonen J (2019). Cell therapy for ischemic stroke: Are differences in preclinical and clinical study design responsible for the translational loss of efficacy?. Ann. Neurol..

[5] Smith DH, Wolf JA, Lusardi TA, Lee VM, Meaney DF (1999). High tolerance and delayed elastic response of cultured axons to dynamic stretch injury. J. Neurosci..

[6] Greer JE, Hanell A, McGinn MJ, Povlishock JT (2013). Mild traumatic brain injury in the mouse induces axotomy primarily within the axon initial segment. Acta Neuropathol..

[7] Marmarou CR, Walker SA, Davis CL, Povlishock JT (2005). Quantitative analysis of the relationship between intra- axonal neurofilament compaction and impaired axonal transport following diffuse traumatic brain injury. J. Neurotrauma.

[8] Okonkwo DO, Pettus EH, Moroi J, Povlishock JT (1998). Alteration of the neurofilament sidearm and its relation to neurofilament compaction occurring with traumatic axonal injury. Brain Res..

[9] Pearn ML (2017). Pathophysiology Associated with Traumatic Brain Injury: Current Treatments and Potential Novel Therapeutics. Cell Mol. Neurobiol..

[10] Ponsford JL (2014). Longitudinal follow-up of patients with traumatic brain injury: outcome at two, five, and ten years post-injury. J. Neurotrauma.

[11] Tang-Schomer MD, Johnson VE, Baas PW, Stewart W, Smith DH (2012). Partial interruption of axonal transport due to microtubule breakage accounts for the formation of periodic varicosities after traumatic axonal injury. Exp. Neurol..

[12] Greer JE, McGinn MJ, Povlishock JT (2011). Diffuse traumatic axonal injury in the mouse induces atrophy, c-Jun activation, and axonal outgrowth in the axotomized neuronal population. J. Neurosci..

[13] Johnson VE, Stewart W, Smith DH (2013). Axonal pathology in traumatic brain injury. Exp. Neurol..

[14] Siedler DG, Chuah MI, Kirkcaldie MT, Vickers JC, King AE (2014). Diffuse axonal injury in brain trauma: insights from alterations in neurofilaments. Front. Cell Neurosci..

[15] Singleton RH, Zhu J, Stone JR, Povlishock JT (2002). Traumatically induced axotomy adjacent to the soma does not result in acute neuronal death. J. Neurosci..

[16] Johnson VE (2013). Inflammation and white matter degeneration persist for years after a single traumatic brain injury. Brain.

[17] Ramlackhansingh AF (2011). Inflammation after trauma: microglial activation and traumatic brain injury. Ann. Neurol..

[18] Ziebell JM, Ray-Jones H, Lifshitz J (2017). Nogo presence is inversely associated with shifts in cortical microglial morphology following experimental diffuse brain injury. Neuroscience.

[19] Felmingham KL, Baguley IJ, Green AM (2004). Effects of diffuse axonal injury on speed of information processing following severe traumatic brain injury. Neuropsychology.

[20] Maas AI, Stocchetti N, Bullock R (2008). Moderate and severe traumatic brain injury in adults. Lancet Neurol..

[21] Hanlon LA, Huh JW, Raghupathi R (2016). Minocycline Transiently Reduces Microglia/Macrophage Activation but Exacerbates Cognitive Deficits Following Repetitive Traumatic Brain Injury in the Neonatal Rat. J. Neuropathol. Exp. Neurol..

[22] Homsi S (2010). Blockade of acute microglial activation by minocycline promotes neuroprotection and reduces locomotor hyperactivity after closed head injury in mice: a twelve-week follow-up study. J. Neurotrauma.

[23] Sanchez Mejia, R. O., Ona, V. O., Li, M. & Friedlander, R. M. Minocycline reduces traumatic brain injury-mediated caspase-1 activation, tissue damage, and neurological dysfunction. Neurosurgery 48, 1393–1399; discussion 1399–1401, 10.1097/00006123-200106000-00051 (2001). 10.1097/00006123-200106000-00051 11383749

[24] Simon DW (2018). Minocycline Attenuates High Mobility Group Box 1 Translocation, Microglial Activation, and Thalamic Neurodegeneration after Traumatic Brain Injury in Post-Natal Day 17 Rats. J. Neurotrauma.

[25] Bye N (2007). Transient neuroprotection by minocycline following traumatic brain injury is associated with attenuated microglial activation but no changes in cell apoptosis or neutrophil infiltration. Exp. Neurol..

[26] Margulies S (2016). Combination Therapies for Traumatic Brain Injury: Retrospective Considerations. J. Neurotrauma.

[27] Siopi E (2011). Minocycline restores sAPPalpha levels and reduces the late histopathological consequences of traumatic brain injury in mice. J. Neurotrauma.

[28] Kovesdi E (2012). Acute minocycline treatment mitigates the symptoms of mild blast-induced traumatic brain injury. Front. Neurol..

[29] Rowe RK, Harrison JL, O’Hara BF, Lifshitz J (2014). Recovery of neurological function despite immediate sleep disruption following diffuse brain injury in the mouse: clinical relevance to medically untreated concussion. Sleep.

[30] Lee SA (2016). Gradient Index Microlens Implanted in Prefrontal Cortex of Mouse Does Not Affect Behavioral Test Performance over Time. Plos One.

[31] Voziyanov, V., Kemp, B. S., Dressel, C. A., Ponder, K. & Murray, T. A. TRIO Platform: A novel low profile *in vivo* imaging support and restraint system for mice. *Frontiers in Neuroscience* **10**, 10.3389/fnins.2016.00169 (2016). 10.3389/fnins.2016.00169 PMC4842766 27199633

[32] Rowe RK, Harrison JL, Ellis TW, Adelson PD, Lifshitz J (2018). Midline (central) fluid percussion model of traumatic brain injury in pediatric and adolescent rats. J. Neurosurg. Pediatr..

[33] Murray TA, Levene MJ (2012). Singlet gradient index lens for deep *in vivo* multiphoton microscopy. J. Biomed. Opt..

[34] Lifshitz, J. Fluid percussion injury model. Vol. 30 369-384 (Humana Press, 2009).

[35] Rowe RK (2018). Novel TNF receptor-1 inhibitors identified as potential therapeutic candidates for traumatic brain injury. J. Neuroinflammation.

[36] Rowe RK (2013). Using anesthetics and analgesics in experimental traumatic brain injury. Lab. Anim..

[37] Rowe RK, Griffiths DR, Lifshitz J (2016). Midline (Central) Fluid Percussion Model of Traumatic Brain Injury. Methods Mol. Biol..

[38] Fujimoto ST (2004). Motor and cognitive function evaluation following experimental traumatic brain injury. Neurosci. Biobehav. Rev..

[39] Harrison JL (2015). Resolvins AT-D1 and E1 differentially impact functional outcome, post-traumatic sleep, and microglial activation following diffuse brain injury in the mouse. Brain, behavior, Immun..

[40] Harrison JL, Rowe RK, O’Hara BF, Adelson PD, Lifshitz J (2014). Acute over-the-counter pharmacological intervention does not adversely affect behavioral outcome following diffuse traumatic brain injury in the mouse. Exp. Brain Res..

[41] Rowe, R. K. *et al*. Acute post-traumatic sleep may define vulnerability to a second traumatic brain injury in mice. *J Neurotrauma*, 10.1089/neu.2018.5980 (2018). 10.1089/neu.2018.5980 PMC6479254 30398389

[42] Rasband, W. S. (1997–2008).

[43] Thevenaz P, Ruttimann UE, Unser M (1998). A pyramid approach to subpixel registration based on intensity. IEEE Trans. Image Process..

[44] Gu Y (2017). Polarity of varicosity initiation in central neuron mechanosensation. J. Cell Biol..

[45] Carter RE (2013). A standard error: distinguishing standard deviation from standard error. Diabetes.

[46] Smith DH, Meaney DF (2000). Axonal Damage in Traumatic Brain Injury. Neuroscientist.

[47] Lifshitz J, Kelley BJ, Povlishock JT (2007). Perisomatic thalamic axotomy after diffuse traumatic brain injury is associated with atrophy rather than cell death. J. neuropathology Exp. Neurol..

[48] Pierce JE, Trojanowski JQ, Graham DI, Smith DH, McIntosh TK (1996). Immunohistochemical characterization of alterations in the distribution of amyloid precursor proteins and beta-amyloid peptide after experimental brain injury in the rat. J. Neurosci..

[49] Hanell A, Greer JE, McGinn MJ, Povlishock JT (2015). Traumatic brain injury-induced axonal phenotypes react differently to treatment. Acta Neuropathol..

[50] Armstrong RC, Mierzwa AJ, Marion CM, Sullivan GM (2016). White matter involvement after TBI: Clues to axon and myelin repair capacity. Exp. Neurol..

[51] Reeves TM, Phillips LL, Povlishock JT (2005). Myelinated and unmyelinated axons of the corpus callosum differ in vulnerability and functional recovery following traumatic brain injury. Exp. Neurol..

[52] Weber MT, Arena JD, Xiao R, Wolf JA, Johnson VE (2019). CLARITY reveals a more protracted temporal course of axon swelling and disconnection than previously described following traumatic brain injury. Brain Pathol..

[53] Siopi E (2012). Evaluation of late cognitive impairment and anxiety states following traumatic brain injury in mice: the effect of minocycline. Neurosci. Lett..

[54] Taylor AN, Tio DL, Paydar A, Sutton RL (2018). Sex Differences in Thermal, Stress, and Inflammatory Responses to Minocycline Administration in Rats with Traumatic Brain Injury. J. Neurotrauma.

[55] d’Avila JC (2012). Microglial activation induced by brain trauma is suppressed by post-injury treatment with a PARP inhibitor. J. Neuroinflammation.

[56] Kielian T (2007). Minocycline modulates neuroinflammation independently of its antimicrobial activity in staphylococcus aureus-induced brain abscess. Am. J. Pathol..

[57] Min Y (2017). Minocycline-Suppression of Early Peripheral Inflammation Reduces Hypoxia-Induced Neonatal Brain Injury. Front. Neurosci..

[58] Yenari MA, Xu L, Tang XN, Qiao Y, Giffard RG (2006). Microglia potentiate damage to blood-brain barrier constituents: improvement by minocycline *in vivo* and *in vitro*. Stroke.

[59] Scott G (2018). Minocycline reduces chronic microglial activation after brain trauma but increases neurodegeneration. Brain.

[60] O’Connor CA, Cernak I, Johnson F, Vink R (2007). Effects of progesterone on neurologic and morphologic outcome following diffuse traumatic brain injury in rats. Exp. Neurol..

[61] Roof RL, Hall ED (2000). Gender differences in acute CNS trauma and stroke: neuroprotective effects of estrogen and progesterone. J. Neurotrauma.

[62] Tucker LB, Burke JF, Fu AH, McCabe JT (2017). Neuropsychiatric Symptom Modeling in Male and Female C57BL/6J Mice after Experimental Traumatic Brain Injury. J. Neurotrauma.

