# Supplementary material for: Longitudinal optical imaging technique to visualize progressive axonal damage after brain injury in mice reveals responses to different minocycline treatments
Source: Sci Rep. 2020 May 8;10:7815. doi: 10.1038/s41598-020-64783-x (PMC7210987; doi:10.1038/s41598-020-64783-x)
Supplement: Supplementary file 1 — Supplementary Information. [file 41598_2020_64783_MOESM1_ESM.pdf]

Supplemental Data

Longitudinal optical imaging technique to visualize progressive axonal damage after brain injury in mice reveals responses to different minocycline treatments

Chelsea D. Pernici[1], Rachel K. Rowe[2-4], P. Timothy Doughty[1], Mahboubeh Madadi[5],
Jonathan Lifshitz[2-4], Teresa A. Murray[1,*]

1 Center for Biomedical Engineering and Rehabilitation Sciences, Louisiana Tech University, Ruston, Louisiana, USA
2 Barrow Neurological Institute at Phoenix Children's Hospital, Phoenix, AZ, USA
3 Department of Child Health, University of Arizona College of Medicine – Phoenix, Phoenix, AZ, USA
4 Phoenix Veterans Affairs Health Care System, Phoenix, AZ, USA
5 Department of Business Analytics, San Jose State University, San Jose, CA, USA

* Corresponding author: Dr. Teresa Murray, PO Box 10157, Ruston, LA, USA 71272-0046, tmurray@latech.edu, bioengineer1@hotmail.com

The following content contains supplemental figures that were referred to in the main article.

Day 60 Images

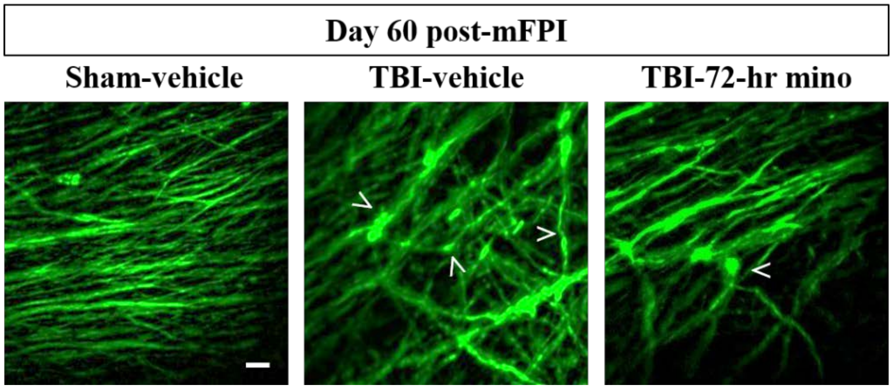

Figure S1. Day 60 post-mFPI. Representative images at Day 60 from sham-vehicle, TBI-vehicle, and TBI-72-hr minocycline (mino) treated mice. At Day 60, none of the TBI-45-min minocycline treated animals had attached head plates. Baseline through Day 30 images for these animals are in Figure 5 of the main text. At Day 60, some axons still have varicosities (white >). Scale bar = 10µm.

Head plate designed to interface with injury hub

The files for printing the head plate[1] and other components of the TRIO in vivo imaging support platform[2] are available on the National Institutes of Health 3D Print Exchange at https://3dprint.nih.gov/discover/3dpx-010720.

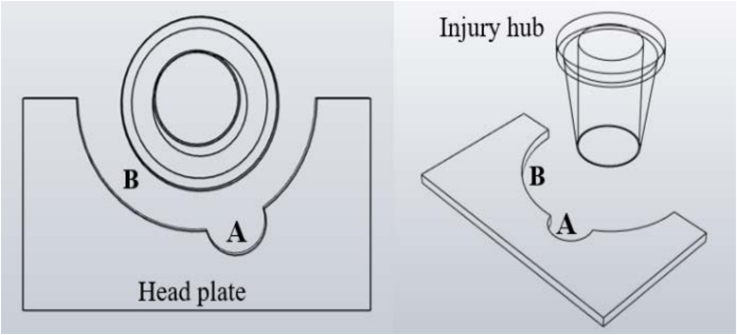

Figure S2. 3D printed head plate for use with an injury hub. The head plate was designed with two cutouts. Cutout **A** allows for easy alignment with the stainless-steel washer portion of the implanted imaging system. Cutout **B** allows for a second surgery in which an injury hub is placed to facilitate fluid percussion injury.

Behavioral test results at Day 60

We describe our methods for the behavioral tests in the last section.

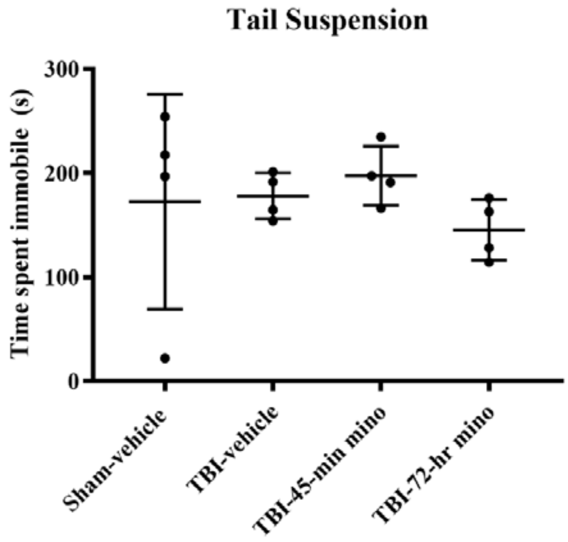

Figure S3. Tail suspension test results. No significant difference was found between treatment groups. Animals spent a similar amount of time immobile while suspended. Data are presented as mean ± SD. Sham-vehicle n=4, TBI-vehicle n=4, TBI-45-min minocycline (mino in data labels) n=4, TBI-72-hr minocycline n=4, ANOVA. ANOVA test power is 0.168. Note: animals were excluded if they climbed their tails during test and one animal was excluded from the TBI-72-hr group for data collection problem.

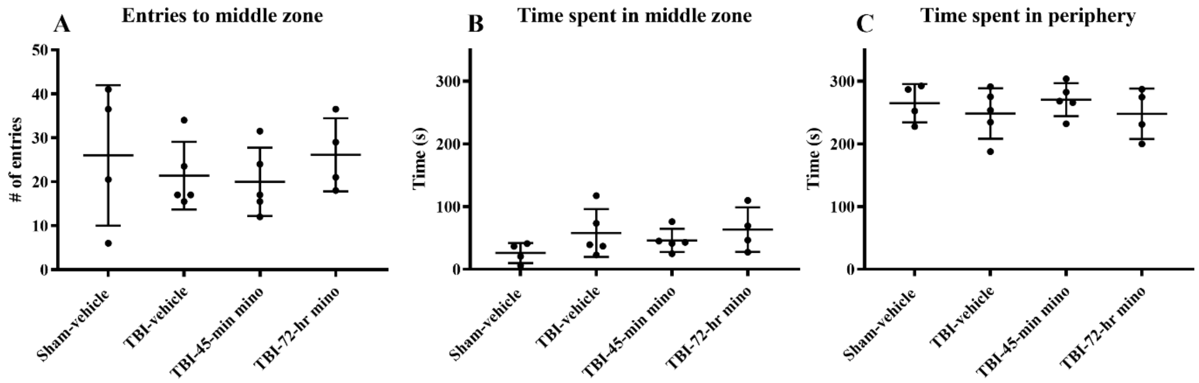

Figure S4. Open field test. No difference was found between treatment groups in **A)** the number of entries into the middle zone ($F_{3,14}$=0.4266, p=0.7370), **B)** the time spent in the center of the testing apparatus ($F_{3,14}$=1.343, p=0.3006), or **C)** the time spend in the periphery of the testing apparatus ($F_{3,14}$=0.501, p =0.687). Sham-vehicle (n=4), TBI-vehicle (n=5), TBI-45-min minocycline (n=5), TBI-72-hr (n=4). Data are presented as mean ± SD, ANOVA with Bonferroni correction. Note: one animal was excluded in the TBI-72-hr minocycline (mino in data labels) group for data collection problem.

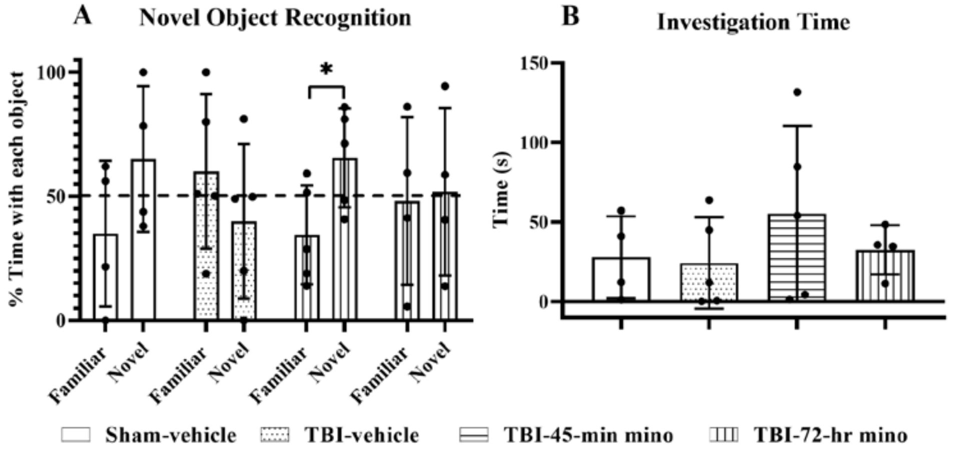

Figure S5. Novel object recognition and discrimination index. A) TBI-45-min minocycline (mino in legend) treated animals spent significantly more time with the novel object as compared to the familiar object (p=0.04). Other groups did not spend a significantly longer amount of time with the novel object ($p<0.05$). Data is represented as mean ± SD. * $p<0.05$, unpaired, two-tailed t-test. **B)** No statistical difference was found between treatment groups in their ability to discern between objects.. Sham-vehicle n=4, TBI-vehicle n=5, TBI-45-min minocycline n=5, TBI-72-hr minocycline n=4. Data are presented as the mean ± SD; ANOVA with Bonferroni correction. Note: one animal was excluded in the TBI-72-hr minocycline group for data collection problem.

Behavioral tests to assess effects of new treatment window for minocycline.

Open Field Test. The open field test was used to assess locomotor activity and anxiety-like behavior. To begin the test, the animal was placed in the middle of a white empty arena (30 cm x 30 cm with 25-cm high walls) and allowed to explore for five min. Movement was recorded by a video camera (iPad, Apple Inc.) positioned directly above the field. Video recordings were analyzed offline using Nodulus Observer. The time spent in the center of the arena (15 cm x15 cm), the time spent in the periphery, and number of entries into the center were recorded[3,4]. One animal's data from the TBI-72-hr minocycline treated group was not analyzed due to recording equipment failure.

Novel Object Recognition. Cognitive impairment was tested using the novel object recognition (NOR) test as previously published[5-7]. On day 60, mice were placed in an open field (30 cm x 30 cm x 25 cm) for a 1 hr habituation period. Mice were removed, and two identical objects were placed in opposing corners of the arena. Animals were placed in the center of the arena with their snouts opposing the objects. Animals were allowed to explore the two identical objects for five min and then they were returned to their home cages. One of the objects was removed and a novel object was put in its place. The animals were placed in the arena immediately after switching the objects and allowed to explore for five min before returning them to their home cages. The task was recorded by an overhead camera and the amount of time spent with the objects was recorded by observers blinded to treatment condition. Investigation of the objects included sniffing, touching, or climbing onto the object while facing the object. However, if an animal climbed onto an object and sniffed the air, this was not included as time investigating the object. One animal's

data from the TBI-72-hr minocycline treated group was not analyzed due to recording equipment failure. Data are presented as time spent investigating each object and as a discrimination index (DI), in which $DI = \frac{T_{novel} - T_{familiar}}{T_{novel} + T_{familiar}}$.

Tail Suspension. The tail suspension task was initiated by securing mice to a beam by the tail using adhesive tape positioned 20 cm from the ground for six min. The first min was used as an acclimation and not included in the analysis. The task was recorded by a camera and the time that the animal was immobile (making no active movement) was calculated offline by blinded observers. Each video was scored by two observers and the time immobile was averaged to reduce variability[8,9]. One animal's data from the TBI-72-hr minocycline treated group was not analyzed due to recording equipment failure. Animals were excluded from the analysis for climbing their tail during the test (TBI-vehicle n = 1; TBI-45-min minocycline n=1).

References

1 Pernici, C. D., Kemp, B. S. & Murray, T. A. Time course images of cellular injury and recovery in murine brain with high-resolution GRIN lens system. *Sci Rep* **9**, 7946, doi:10.1038/s41598-019-44174-7 (2019).

2 Voziyanov, V., Kemp, B. S., Dressel, C. A., Ponder, K. & Murray, T. A. TRIO Platform: A novel low profile in vivo imaging support and restraint system for mice. *Frontiers in Neuroscience* **10**, doi:10.3389/fnins.2016.00169 (2016).

3 Rowe, R. K. *et al.* Acute post-traumatic sleep may define vulnerability to a second traumatic brain injury in mice. *J Neurotrauma*, doi:10.1089/neu.2018.5980 (2018).

4 Rowe, R. K. *et al.* Aging with Traumatic Brain Injury: Effects of Age at Injury on Behavioral Outcome following Diffuse Brain Injury in Rats. *Dev Neurosci* **38**, 195-205, doi:10.1159/000446773 (2016).

5 Rowe, R. K., Harrison, J. L., Ellis, T. W., Adelson, P. D. & Lifshitz, J. Midline (central) fluid percussion model of traumatic brain injury in pediatric and adolescent rats. *J Neurosurg Pediatr* **22**, 22-30, doi:10.3171/2018.1.PEDS17449 (2018).

6 Harrison, J. L. *et al.* Resolvins AT-D1 and E1 differentially impact functional outcome, post-traumatic sleep, and microglial activation following diffuse brain injury in the mouse. *Brain Behav Immun* **47**, 131-140, doi:10.1016/j.bbi.2015.01.001 (2015).

7 Rowe, R. K., Harrison, J. L., O'Hara, B. F. & Lifshitz, J. Recovery of neurological function despite immediate sleep disruption following diffuse brain injury in the mouse: clinical relevance to medically untreated concussion. *Sleep* **37**, 743-752, doi:10.5665/sleep.3582 (2014).

8 Can, A. *et al.* The tail suspension test. *J Vis Exp*, e3769, doi:10.3791/3769 (2012).

9 Steru, L., Chermat, R., Thierry, B. & Simon, P. The tail suspension test: a new method for screening antidepressants in mice. *Psychopharmacology (Berl)* **85**, 367-370 (1985).
